# Benign Ectopic Thyroid Tissue in the Neck: A Case Report of a Rare Finding

**DOI:** 10.7759/cureus.7172

**Published:** 2020-03-03

**Authors:** Chrispin O Otondi, Frederick D Cason, Mark Kranc, Abdul Waheed

**Affiliations:** 1 General Surgery, Hospital Corporation of America (HCA) Healthcare/University of South Florida (USF) Morsani College of Medicine Graduate Medical Education (GME): Regional Medical Center Bayonet Point, Hudson, USA; 2 Surgical Oncology, San Joaquin General Hospital, Stockton, USA; 3 Pathology, Oak Hill Hospital, Brooksville, USA; 4 Surgery, Hospital Corporation of America (HCA) West Florida Division, Tampa, USA

**Keywords:** ectopic thyroid tissue, cervical, benign, thyroidectomy, lymphoid tissue

## Abstract

Ectopic thyroid tissue (ETT), though an uncommon finding, is prone to be clustered along the midline in the neck and rarely it shows up as a lateral neck mass. Whenever the ETT is discovered in unusual places, the possibility of malignancy is higher, and rarely a benign variant. We present a 71-year-old female with a past history of hypertension, hypercholesteremia, and thyroid nodules presented to the physician’s office complaining of an unusual swelling in the right side of a neck. The physical examination revealed a rubbery, non-tender, mobile, dominant mass in the right upper neck at the jugulodiagastric region in the upper anterior cervical triangle. Ultrasonography (USG) and computed tomography (CT) of the neck strongly suggested the benign characteristics of the mass. The postoperative histological examination of the specimen was indicative of benign thyroid tissue with no metastatic potential and no lymphoid tissue confirming the diagnosis of ETT. To better understand the clinical, pathological, and radiological nature of this rare disease, we present a rare case of ETT in the lateral cervical area which was resected.

## Introduction

The ectopic thyroid tissue (ETT) is a rare phenomenon with an overall prevalence of 1 in 100,000-300,000 in general population, and 1 in 4,000-8,000 in patients with a pre-existing thyroid disorder [1, 2]. ETT is an unusual condition, generally resulting because of problems of embryologic growth and additionally due to the unforeseen post-operative migration of normal thyroid cells. Likewise, based on the location of the mass in the lateral area and its possible mechanism of the development, Rosai further classified the ETT into six various categories [3, 4]. When abnormal thyroid tissue is identified, the most typical location is along the midline beginning at the base of the tongue [5, 6]. Nevertheless, extremely few published case reports describe ETT in various other places of the body, including the abdominal organs, pelvis, axillary region and also the thoracic cavity [6, 7]. Moreover, clinically, most of the patients with the lateral cervical ETT, present with a painless and mobile mass [7].

Furthermore, in individuals with a distinct thyroid mass, the ETT in aberrant locations considerably elevates the suspicion of metastatic thyroid cancer [1, 8]. In these patients, a proper preoperative diagnostic workup consists of ultrasonography (USG), CT of the neck, and fine-needle aspiration (FNA) for cytology [1, 9]. These imaging modalities will certainly not just distinguish the histological component of ETT as benign vs. malignant but may aid in recognizing the embryonic vs. migratory root causes of the mass [1, 10]. Once the appropriate diagnosis is established curative surgical resection offers the best possible treatment. The current case report is a valuable addition to the limited available literature on this rare condition.

## Case presentation

A 71-year-old female with a basal metabolic index (BMI) of 27.03 kg/m^2^ and a history of hypertension, hypercholesteremia, and the thyroid nodules presented to the surgeon's office upon a referral from her primary care with a progressively enlarging mass in the right side of her neck. She further stated that several years previously she had a hysterectomy, sinus surgery, left knee surgery, breast biopsy, and a neck lump biopsy. She refutes any drug allergy, tobacco use, and alcohol consumption history. Furthermore, she had normal thyroid function studies.

**Figure 1:**
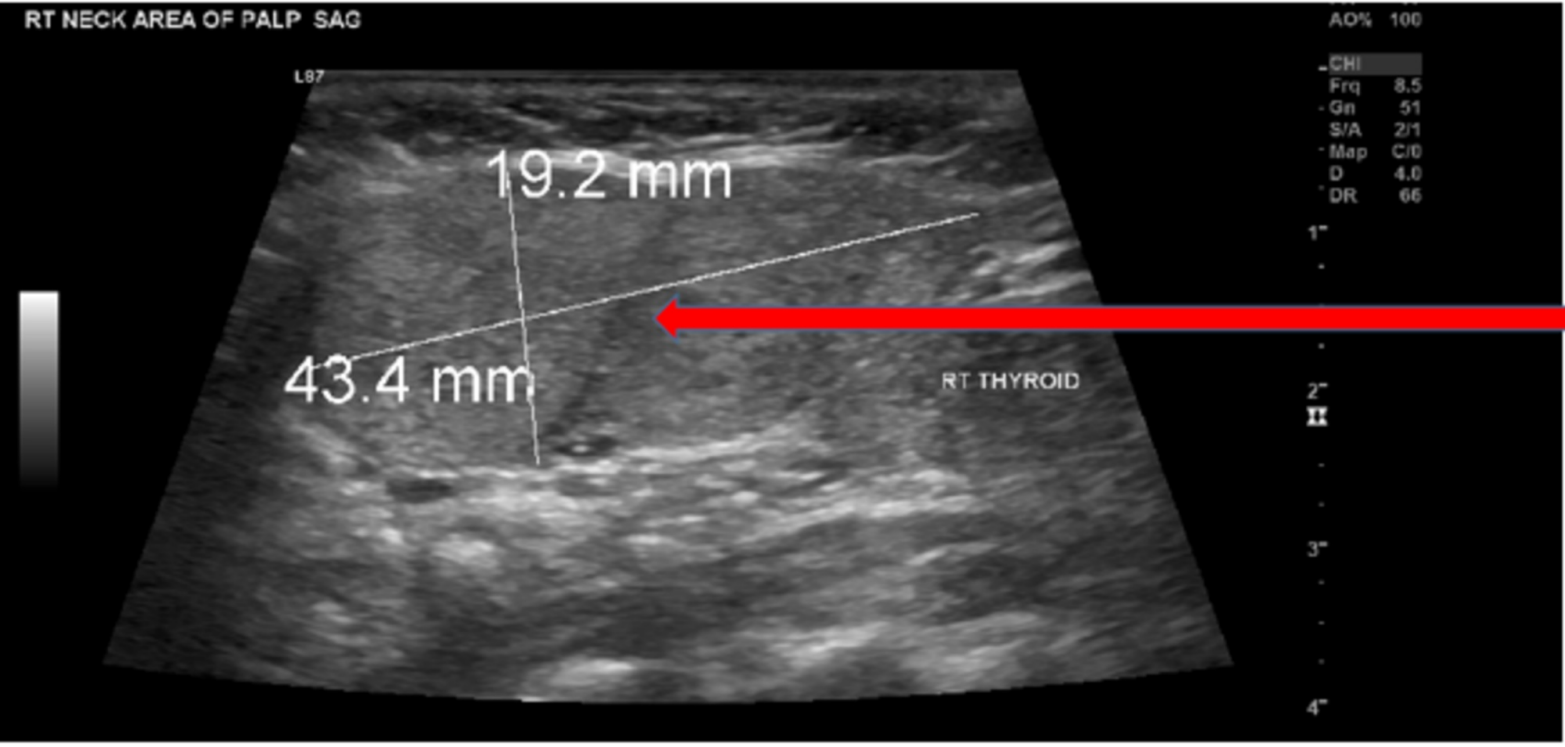
Ultrasound image of the neck Ultrasound image of the neck demonstrating the location of the ectopic thyroid tissue (Red arrow).

**Figure 2:**
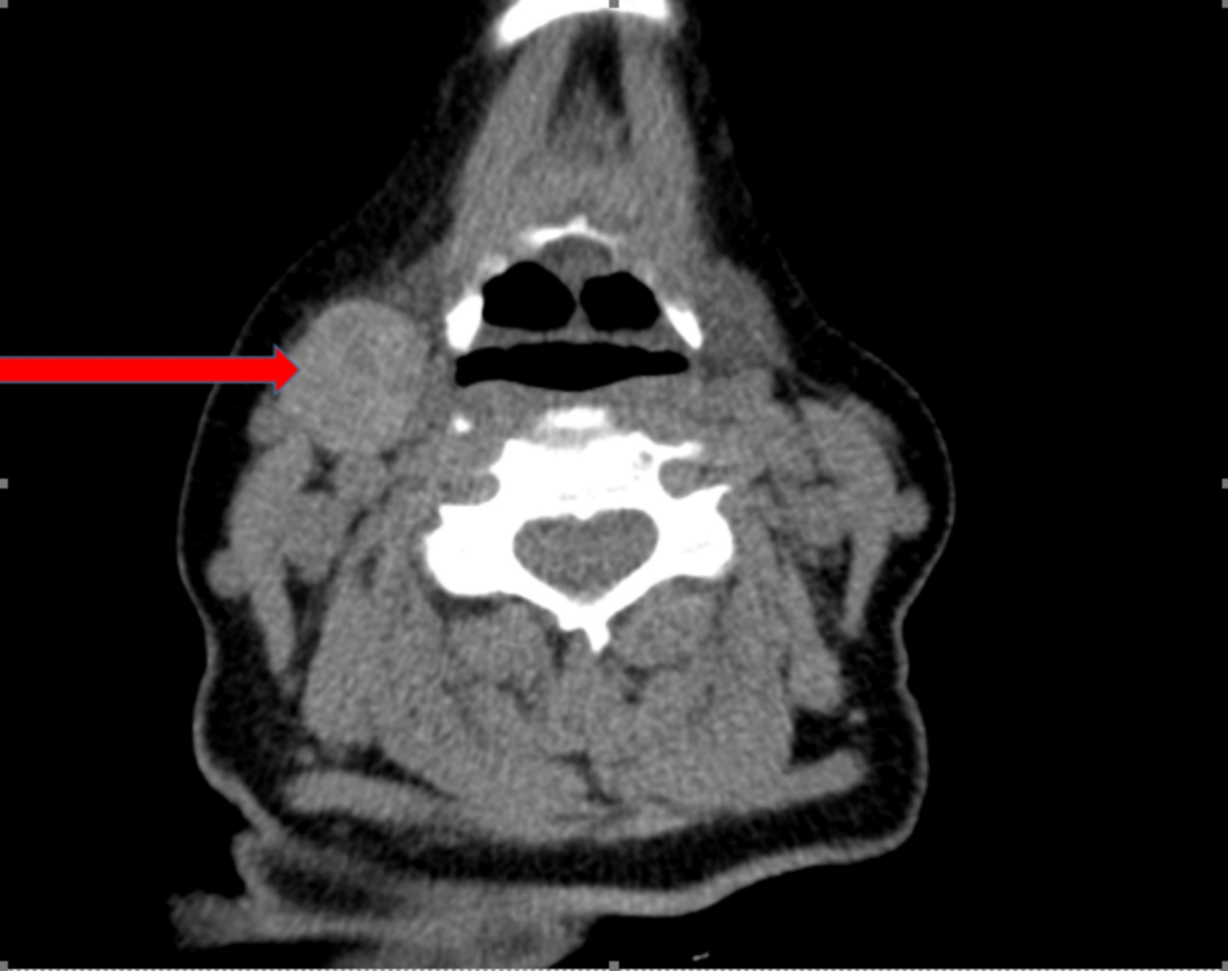
Computed tomography of the neck Computed tomography of the neck demonstrating the location of the ectopic thyroid tissue (Red arrow).

The patient had a USG of the neck showing multi-nodular goiter and a large exophytic 4.3 cm mass near the upper pole of the right thyroid lobe which was not present on a previous study performed a few years earlier (Figure 1). The study revealed two nodules in the right lobe of the thyroid gland measuring 1.1 cm and 1.7 cm, respectively. These were given a Thyroid Imaging Reporting and Data Systems (TIRADS) score of 3. Also, in the left thyroid lobe, the USG of the neck detected two nodules measuring 1.3 cm and 1.8 cm, respectively. A CT scan of the neck was also performed suggesting that the mass in the right upper neck was separate from the thyroid gland and may be an enlarged lymph node (Figure 2). She had undergone previous fine-needle aspiration (FNA) of the right neck mass for cytology and was also benign.

At the initial presentation, she was afebrile (Temperature = 98.5°F) with a blood pressure of 162/78 mmHg, a heart rate of 68 beats/minute, and oxygen saturation (SpO2) of 100%. On physical examination, there was a dominant mass in the right upper neck below the jugulodiagastric region in the anterior cervical triangle that was mobile, soft, rubbery, smooth, and non-tender. The mass did not appear to be attached to the thyroid gland on the clinical examination. The thyroid gland was nodular and minimally enlarged. There was no cervical lymphadenopathy noted in the left neck or beyond the apparent large upper right neck mass. The trachea was normal and midline and no apparent vascular abnormalities were noted.

Based on the physical examination and preoperative diagnostic workup, a decision was made to perform a total thyroidectomy with intraoperative neural monitoring and excision and biopsy of a right neck mass if cancer was to be identified, the next step was to be central and right modified neck dissection. In the operating room, a transverse neck incision was made just above the suprasternal notch.

**Figure 3:**
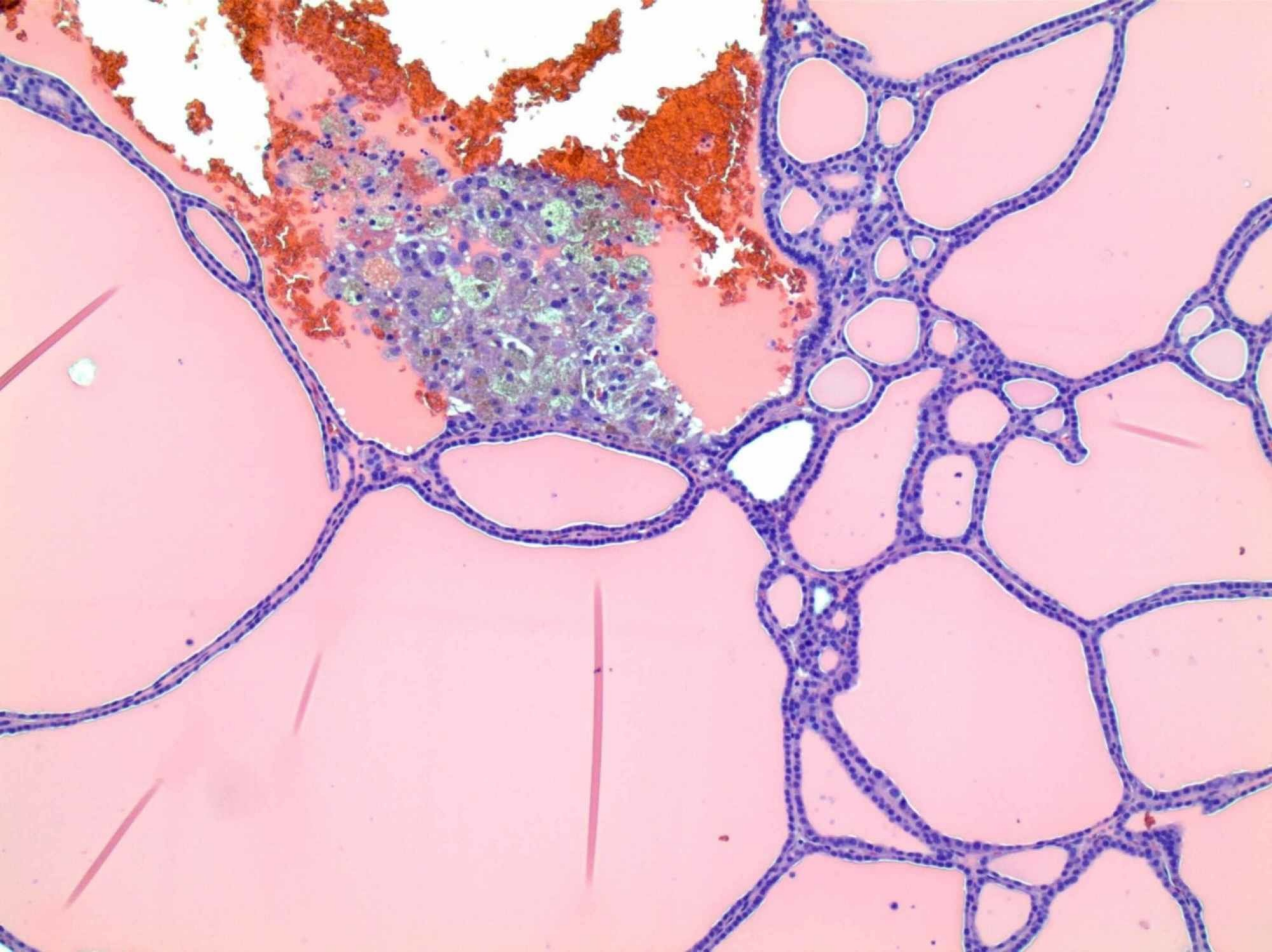
Microscopic picture of the tissue obtained during the surgery Normal appearing thyroid follicles without malignant cells.

A separate incision was made over the visible and palpable mass in the upper right neck. It was enucleated and submitted to the pathologist for touch prep and frozen section histology. The mass measured about 3.9 cm in length and 2 cm in diameter. The pathologist reported that no lymphoid tissue was seen in the mass, and it was purely benign thyroid tissue (Figure 3).

**Figure 4:**
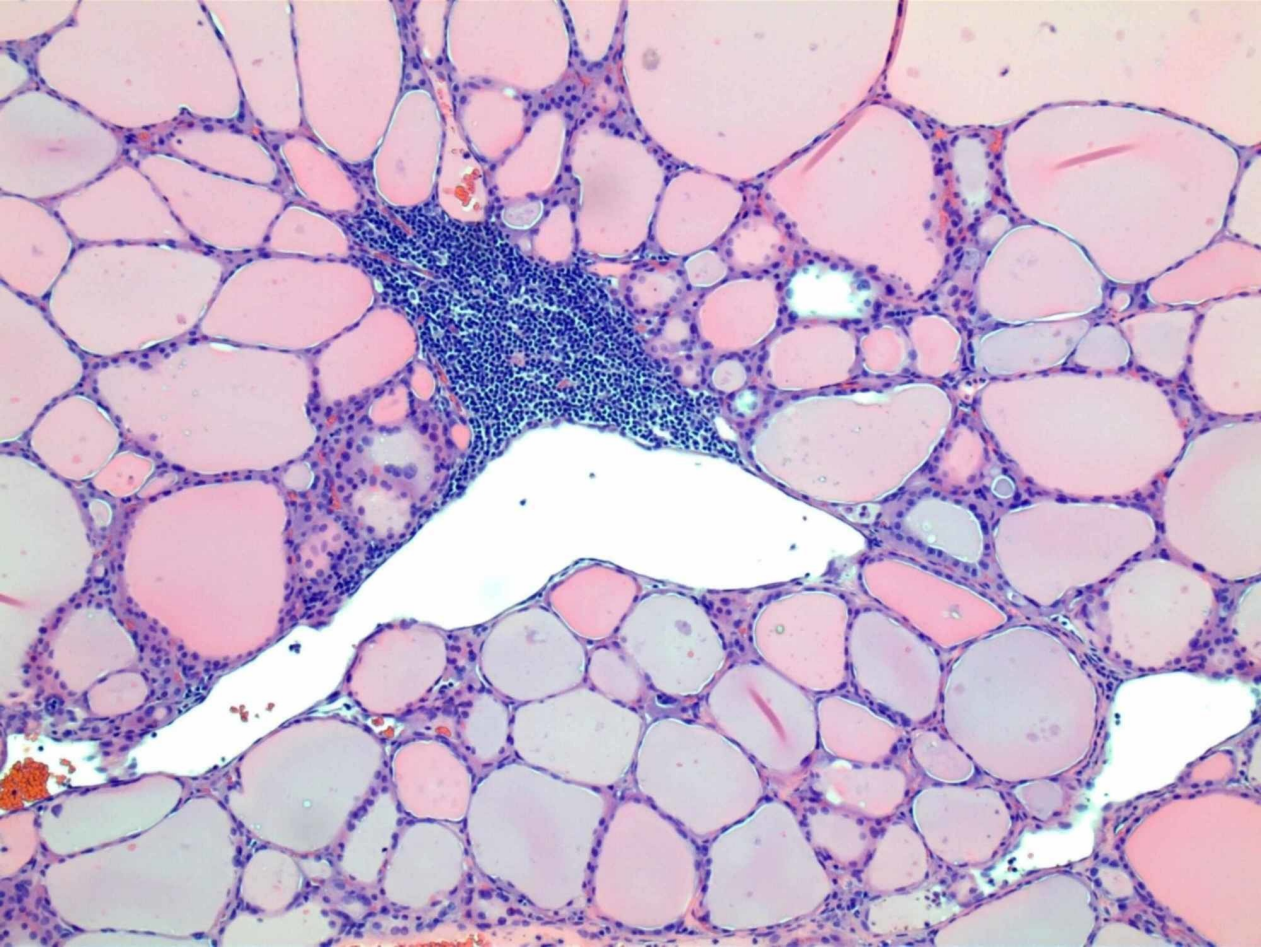
Microscopic picture of the tissue obtained during surgery Normal appearing thyroid follicles without malignant cells.

A total thyroidectomy was completed for the multinodular thyroid goiter. Lymph nodes surrounding the thyroid were submitted for touch prep and frozen section to determine whether there was metastatic thyroid carcinoma. The pathologist did not recognize any malignancy, and it appeared to be an ETT. Post-operative pathological specimen assessment revealed no metastatic foci in any specimens. The specimen retrieved from the right lateral cervical area only contained benign multi-nodular thyroid parenchyma (Figure 4).

## Discussion

ETT is an unusual anomaly. Central neck hypertrophic thyroid tissue (Lingual thyroid) was first reported by Hickman in 1869 when his team discovered this unusual anomaly in a newborn baby that died secondary to the compressive effects of the hypertrophic tissue [11]. Although ETT is rarely reported, the published data suggest that this might be attributed due to irregularities in the embryologic growth, unorganized differentiation, as well as migration of the thyroid cells. However, thyroid ectopy needs to be constantly distinguished from metastatic papillary thyroid cancer, seeding of thyroid tissue during surgical treatment, thyroid cells occurring in a teratoma, as well as local tissue metaplasia [12].

To better recognize the underlying root cause of ETT, it is crucial to understand the fundamental embryological development of the thyroid gland and the thyroglossal duct. Developmentally, the thyroglossal duct usually obliterates around the 40th day of gestation. The ectopic location may best be clarified by the failure of obliteration of the thyroglossal duct which explains the mid-cervical distribution of the thyroglossal duct remnant [12]. However, there are few cases reported as the location of the ETT in extremely unusual places including, but not limited to, the lateral cervical area, axilla, heart, stomach, trachea, small intestine, gallbladder, and ovaries [13, 14]. ETT in the lateral cervical region has mainly been connected to the impaired movement of the lateral thyroid anlagen, the displacement of the median thyroid anlage, thyroid tissue seeding during previous thyroid surgery, and thyroid carcinoma metastasis. Moreover, the finding of in situ differentiation is the most common attributing factors for the ETT in unusual places such as ophthalmic ectopic thyroid tissues [12].

In addition, the available published literature attributes a likely root cause of ETT to the possible met of the malignant thyroidal cells in the aberrant area within the neck. The literature related to the presence of thyroid tissue in the neck lateral to the jugular vein is controversial in terms of differentiating ETT as being a benign condition or metastasis disease [12]. In our 71-year-old patient, there is no definite explanation of the most likely source of the aberrant lateral thyroid tissue. In this age group, as opposed to the findings in other reports where patients were younger, there are no viable explanations related to embryonic remnants, making our case extremely rare.

Histologically, the criterion to distinguish between a primary benign lesion and malignant neoplastic growth of ETT remains the same as used to diagnose the primary tumors. For instance, in order to detect the follicular variant of thyroid carcinoma, the recognition of vascular invasion, capsular invasion, and hematogenous metastasis are the diagnostic features. Likewise, the papillary variant of thyroidal carcinoma is identified by the presence of Psammoma bodies, nuclear contour abnormalities, the presence of grooves, and the ground-glass appearance [13].

The definite diagnosis of lateral cervical ETT is based on tissue biopsy. There are no clinical, non-pathological laboratory tests or imaging parameters that may assist in determining the benign or malignant nature of ETT. Although the USG and CT scan of the neck may describe the consistency, location, and borders of a neck mass, these tests cannot definitively provide the diagnosis without tissue biopsy [15]. FNA biopsy may be suggestive of a benign or malignant lesion, but it is frequently indeterminate. In our case, the preoperative imaging suggested the right neck mass to be of a lymphoid origin. But the definitive diagnosis was made by the tissue biopsy.

Approaches to the treatment of cervical ETT depend on factors such as metastasis, size of the lesion, the presence or absence of the local symptoms, patient’s age, thyroid gland functional status, the uncertainty of the diagnosis, and patient’s comorbidities [13-15]. In cases of histologically normal lateral cervical involvement, the surgical excision of the mass alone is satisfactory treatment. In the case of the abnormal thyroid nodule or hyperthyroidism synchronous with ectopic tissue, the thyroidectomy and excision of the ETT may offer the optimal treatment [11, 15]. To the best of the author’s knowledge, we did not find cases of medical treatment with thyroid hormone suppression or radioactive iodine in the medical literature. In rare cases, the radical neck dissection may be needed if the intraoperative specimen evaluation is not conclusive for benign histology, and there is a substantial tumor extension into the adjacent areas [15].

## Conclusions

In elderly patients, the presence of ETT in the lateral cervical regions is rare. It should be suspected in patients with a previous history of thyroid surgery or tissue biopsy. The seeding of cells from disrupted thyroid tissue may be the possible mechanism. The preoperative diagnostic tests, including USG and CT scan of the neck, may provide anatomic details but do not clarify the diagnosis. Histopathologic specimen evaluations are the cornerstone of the diagnostic confirmation. The definitive treatment rest is the surgical excision of the abnormality.
